# Betaine attenuates LPS-induced downregulation of Occludin and Claudin-1 and restores intestinal barrier function

**DOI:** 10.1186/s12917-020-02298-3

**Published:** 2020-03-04

**Authors:** Jingtao Wu, Caimei He, Jie Bu, Yue Luo, Shuyuan Yang, Chengyan Ye, Silei Yu, Binsheng He, Yulong Yin, Xiaoping Yang

**Affiliations:** 1 Key Laboratory of Study and Discovery of Small Targeted Molecules of Hunan Province, Department of Pharmacy, School of Medicine, Hunan Normal University, Changsha, Hunan 410013 People’s Republic of China; 2 Changsha Medical University, Changsha, 410219 Hunan People’s Republic of China; 3 Institute of Subtropical Agriculture, Chinese Academy of Science, Changsha, 410125 People’s Republic of China

**Keywords:** Betaine, Tight junctions, Transepithelial electrical resistance, Intestinal porcine epithelial cells

## Abstract

**Background:**

The intestinal epithelial barrier, which works as the first line of defense between the luminal environment and the host, once destroyed, it will cause serious inflammation or other intestinal diseases. Tight junctions (TJs) play a vital role to maintain the integrity of the epithelial barrier. Lipopolysaccharide (LPS), one of the most important inflammatory factors will downregulate specific TJ proteins including Occludin and Claudin-1 and impair integrity of the epithelial barrier. Betaine has excellent anti-inflammatory activity but whether betaine has any effect on TJ proteins, particularly on LPS-induced dysfunction of epithelial barriers remains unknown. The purpose of this study is to explore the pharmacological effect of betaine on improving intestinal barrier function represented by TJ proteins. Intestinal porcine epithelial cells (IPEC-J2) were used as an in vitro model.

**Results:**

The results demonstrated that betaine enhanced the expression of TJ proteins while LPS (1 μg/mL) downregulates the expression of these proteins. Furthermore, betaine attenuates LPS-induced decreases of TJ proteins both shown by Western blot (WB) and Reverse transcription-polymerase chain reaction (RT-PCR). The immunofluorescent images consistently revealed that LPS induced the disruption of TJ protein Claudin-1 and reduced its expression while betaine could reverse these alterations. Similar protective role of betaine on intestinal barrier function was observed by transepithelial electrical resistance (TEER) approach.

**Conclusion:**

In conclusion, our research demonstrated that betaine attenuated LPS-induced downregulation of Occludin and Claudin-1 and restored the intestinal barrier function.

## Background

Intestinal barrier, a single layer of cells located in the inner wall of the intestine, is mainly composed by the enterocyte membranes and the TJs between enterocytes [1]. The integrity of the intestinal barrier is critical for the health of humans and animals. Destruction of the intestinal barrier give rise to increased intestinal permeability, which in turn accelerates the translocation of pathogens or other harmful substances to the blood stream [2]. This disruption will also contribute to the progress of the necrotizing enterocolitis (NEC) and inflammatory bowel disease (IBD) [3, 4]. Thus, protecting the completeness of the intestinal barrier is an excellent strategy to prevent the progress of both IBD and NEC. Intestinal epithelial cells are closely bound by tight junctional complexes between the cells, which regulates the permeability of adjacent cells and is critical to the integrity of the epithelial barrier [5, 6], and one kind of the essential complex is TJ proteins, Claudin-1 and Occludin for instance.

Betaine, also called glycine betaine, is a kind of natural compound which is wildly distributed in organic organisms [7] and can be easily obtained from the plant beet. It is reported that betaine, as an effective organic osmolyte plays an important role in regulating cells’ adaptation to adverse osmotic environment [8]. Besides, betaine also has the activities of anti-inflammatory and can improve intestinal function [9]. Some researchers find that betaine has osmotic protection properties that help protecting the proteins and enzymes of intestinal cells from environmental stress. Inclusion of betaine in the diet have a beneficial effect on relieving physical reactions to heat stress in both poultry and growing-finishing pigs [10, 11]. However, the specific mechanisms regarding how does it improve the intestinal function remains elusive.

Lipopolysaccharide (LPS), an inflammatory stimulator, can destroy the intestinal barrier [12], which lead to the increase of intestinal permeability, the destroy of TJ proteins and the inflammation of the gut [13–16]. Caco-2, the human colon cancer cell line is commonly used as an intestinal cell model. However, this approach has been questioning due to its abnormal characters including rapid growth and proliferation properties. Recently, differentiated IPEC-J2 cell line is recognized as a better cellular model to investigate the role of the intestinal barrier [17]. Thus, in this study, IPEC-J2 cell was applicated as a cellular model to evaluate the effects of betaine in intestinal barrier function with the treatment of betaine alone or the combination treatment with LPS. The purpose of this study is to explore the mechanistic effect of betaine on improving intestinal barrier function, based on the results of WB, RT-PCR, TEER and Immunofluorescence.

## Results

### Effects of betaine on the expression of tight junction protein in IPEC-J2 cells

**Fig. 1:**
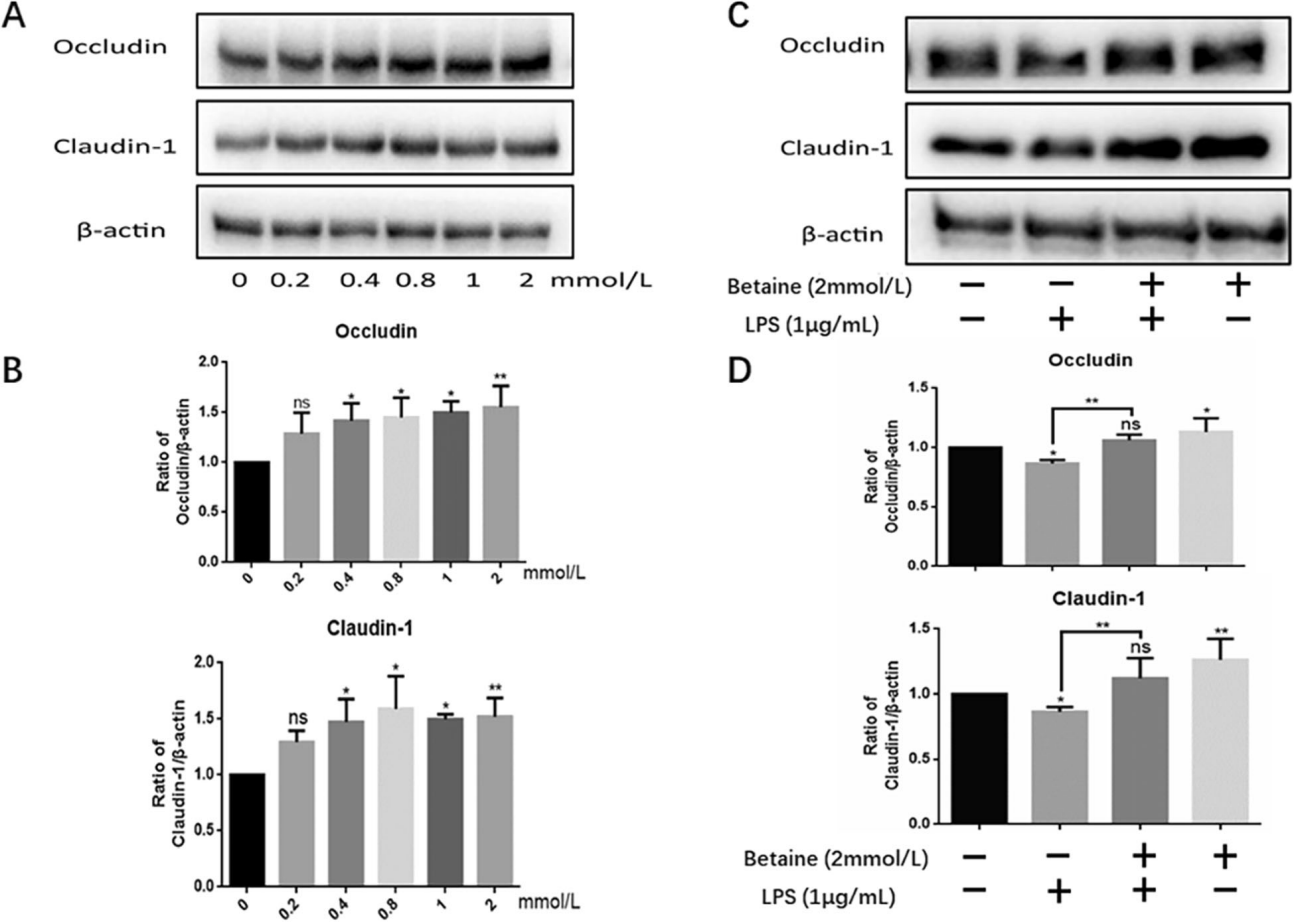
West blot results of samples. **a** showed the results of western blot about Occludin and Claudin-1. After treated for 24 h, various concentrations of betaine (0-2 mmol/L) improve the expression Occludin and Claudin-1 in IPEC-J2 cells. **c** confirms that LPS down-regulate the protein levels of Occludin and Claudin-1, while betaine totally attenuated the down-regulation and recovered to a normal level. **b** and **d** were the statistical graphs of the density ratios of different proteins to β-actin calculated by Image J and analyzed by Graphpad Prism6. All the results were repeated four times (*n* = 4, *P < 0.05, **P < 0.01 compared with control)

First, cells were treated with different concentrations of betaine (0-2 mmol/L) in order to determine its effect on TJ proteins. The results (Fig. 1a-b) clearly demonstrated that different concentrations of betaine can enhance the expression of Occludin and Claudin-1 with optimized concentration of 2 mmol/L. From the results we decided to choose the concentration of 2 mmol/L for the subsequent experiments. Next, we used 1 μg /ml LPS to treat the cells, the same dose as we did before [18]. Consistent with our previous report [18], 1 μg/ml LPS reduced the protein expression of Occludin and Claudin-1. More importantly, the protein expression levels of Occludin and Claudin-1 were significantly recovered with the combination treatment of betaine and LPS compared with LPS alone.

### Effects of betaine on gene expression of Claudin-1

**Fig. 2:**
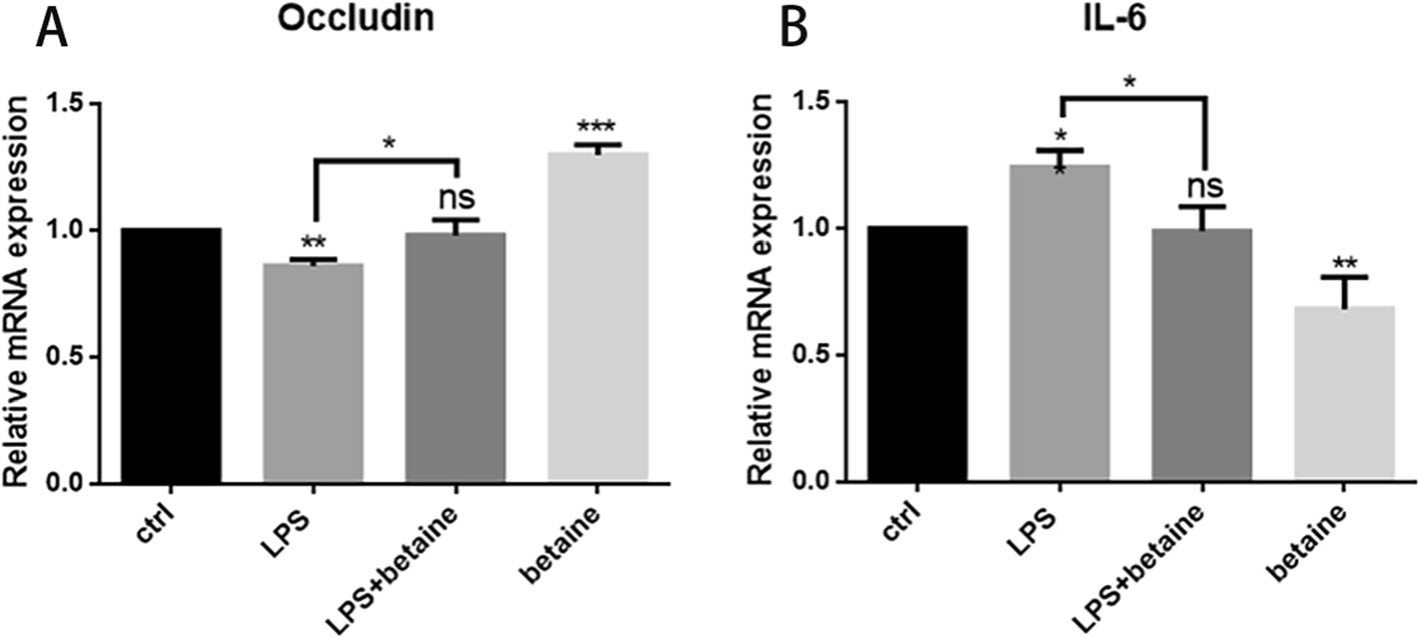
RT-PCR results of samples. **a** confirmed that betaine(2 mmol/L, treated for 4 h)can recover the down-regulation of Occludin induced by LPS(1 μg/mL), and enhanced the mRNA expression of Occludin. **b** proved that LPS induced inflammation while co-treatment of betaine and LPS make the IL-6expression drop to normal levels. Besides, betaine alone will lower the IL-6 expression. All the results were repeated three times (*n* = 3, *P < 0.05, ** *P* < 0.01, ***P < 0.001 compared with control)

To further confirmed the effects of betaine, RT-PCR was used to determine the mRNA expression level. We treated IPEC-J2 for 4 h with ctrl (the control group), LPS (1 μg/mL), LPS (1 μg/mL) + betaine (2 mmol/mL) and betaine (2 mmol/mL) four groups. As shown in Fig. 2, LPS reduced the mRNA expression level of Occludin and increased the expression of IL-6, indicating that LPS destroyed TJs and induced inflammation. And the expression of IL-6 and Occludin of LPS + betaine group was decreased almost to the same as ctrl, indicating that betaine could enhance the mRNA expression of Occludin and exert the function of anti-inflammation.

### Effects of betaine on cell morphology

**Fig. 3:**
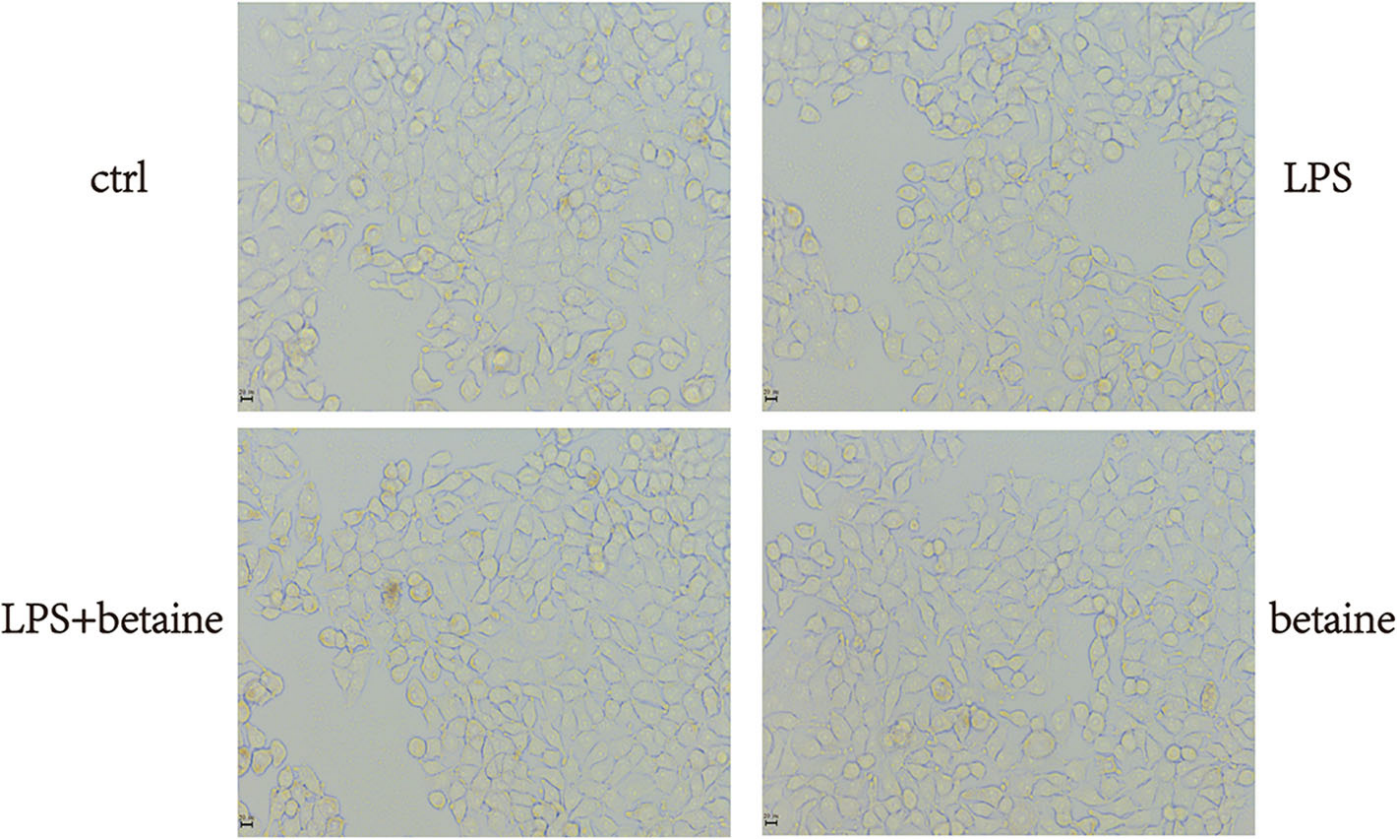
Cellular morphology of four groups with different treatments. There was no obvious difference among four groups, all the cells looked normal and intact

Based on the above results, we are interested in if there are any change of the cell morphology after different treated. Therefore, we culture the cells and then treated with ctrl, LPS, LPS + betaine and betaine alone for 24 h. The culture medium was poured out and then the cells were soaked in PBS and taken photos under the microscope. It could be observed from the Fig. 3 that after different treatments, the cell morphology didn’t have an obvious change.

### Immunofluorescence to detect the expression and localization of Claudin-1

**Fig. 4:**
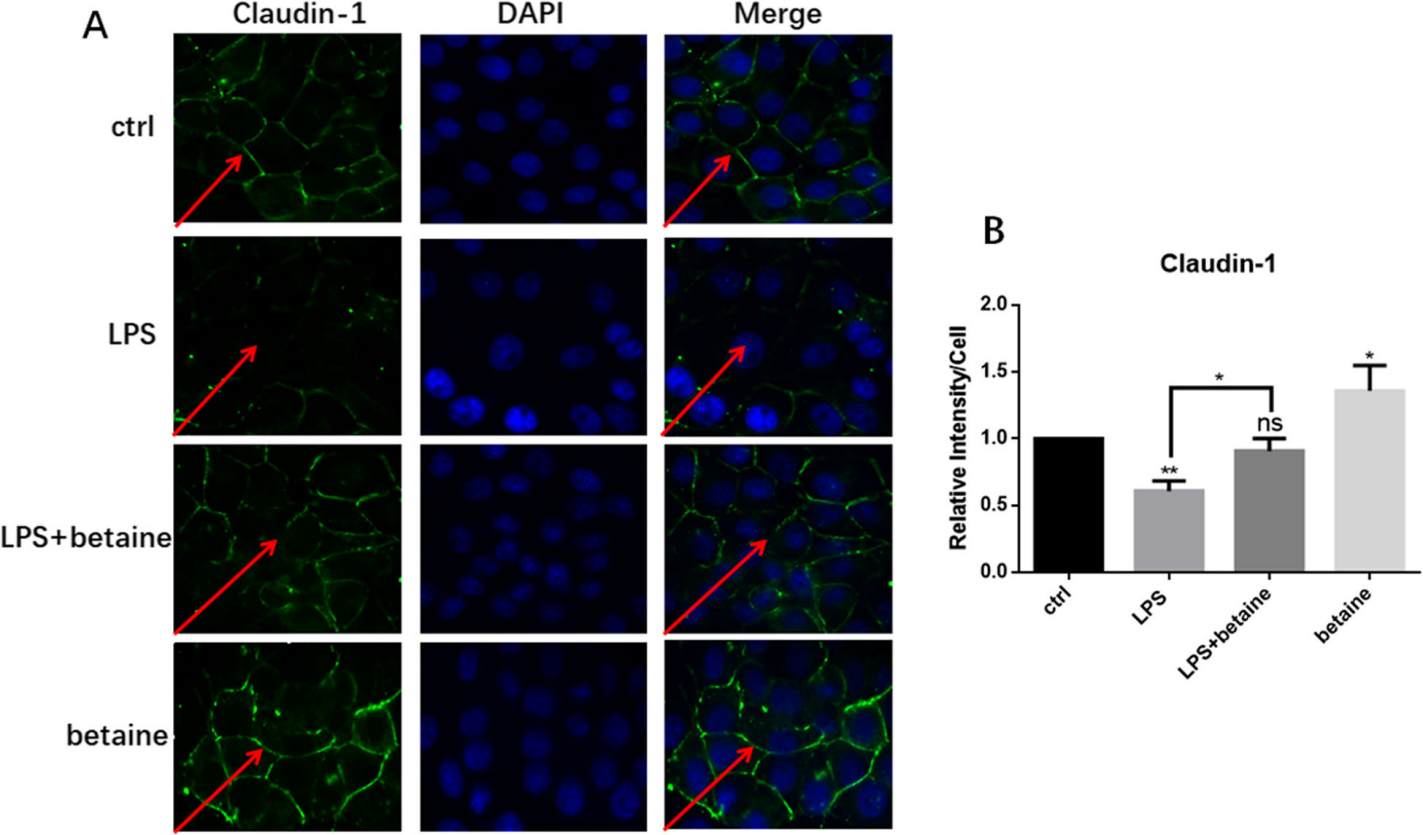
Immunofluorescence results of four groups with different treatments. **a** showed the result of immunofluorescence localization of the TJ protein Claudin-1. The TJ protein of control processing group was in good connection while the LPS treatment group was seriously destroyed. The LPS and betaine combined treatment group was much better than the LPS treatment group and nearly the same as the control group. Betaine alone treatment group had the best expression of Claudin-1. **b** was the semi-quantitative analysis of fluorescence intensity. All the results were repeated three times (*n* = 3, *P < 0.05, ** *P* < 0.01, compared with control)

To further observe and verify the effect of betaine on TJ proteins, immunofluorescence was used since the results obtained from the immunofluorescence microscope are more visualized. After different processing, we observed the localization and expression of TJ protein Claudin-1 (Fig. 4**)**. The protein of ctrl is complete and well-connected while LPS destroyed it. When co-treated with betaine it was recovered, and excitingly betaine alone processing team has the best expression, which proved that betaine can enhance the expression of TJ protein.

### Betaine increases the TEER in LPS-induced IPEC-J2 cells

**Fig. 5:**
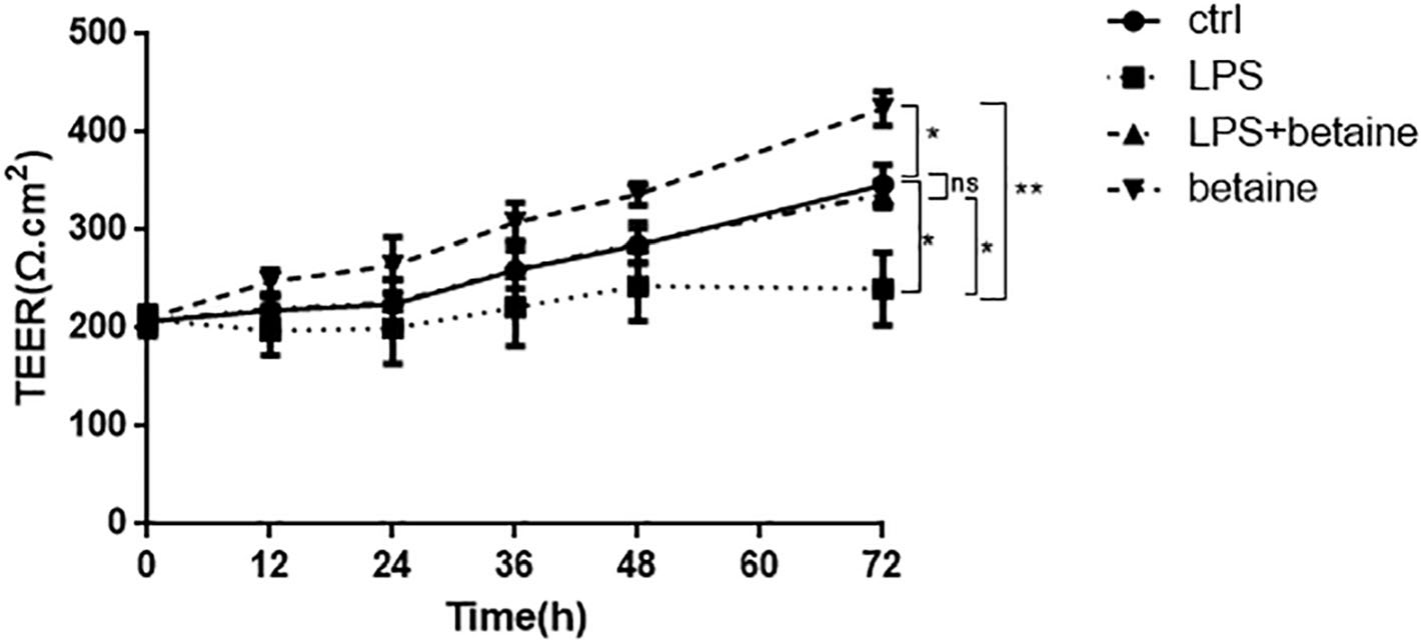
TEER results of four groups with different treatments. The result of TEER measurement showed that LPS(1 μg/mL) decreased the TEER, while combined with betaine, the TEER was relieved to normal. And betaine alone increased the TEER and all the effects continued to 72 h. Noticeably, all the TEER value in 0 h were very close and didn’t have a big margin of error. All the results were repeated three times (Data are expressed by means ± SD, *n* = 3, *p < 0.05 ** *P* < 0.01, comparison between various groups by two-way ANOVA)

Differentiated IPEC-J2 cell was used to investigate the role of the intestinal barrier function as a cellular model, and TEER was used to measure the integrity of intestinal barrier function and as an indicator of intestinal barrier function. Previous studied has proved that LPS increased the permeability of the gut and disordered the intestinal barrier function. However, whether betaine plays any role in LPS-induced intestinal permeability variation remains unknown. As shown in Fig. 5, LPS caused the decline of TEER in 12 h and keep it to 72 h. In contrast, betaine treated alone group increased the TEER value obviously, and the TEER of LPS and betaine combined team was almost the same as ctrl, which fully proved that betaine is able to keep the intestinal permeability and improve intestinal barrier function.

## Discussion

Betaine is a stable and non-toxic natural compound [19] and is widely distributed in organisms such as bacteria and mammals [20], especially abundant in the plant beet. Betaine is an elementary biochemical molecule which participates in the methionine/homocysteine cycle [21, 22] and acts as a methyl donor during methyl-transformation [23, 24]. It is also an osmotic pressure protector which is important to maintain the function of intestinal tissues. There are lots of credibility evidences show that betaine also have the function of anti-inflammation and improving intestinal barrier function [25]. Yang et al [26] found that betaine alleviates monocrotaline-induced pulmonary hypertension in rat by restraining the inflammatory response, such as down-regulating the NF-κB signaling pathway or inhibiting the inflammatory reaction. Olli K et al [27] demonstrated that betaine have the function to reduce the expression of inflammatory adipokines. Besides, Wang et al [8] found that betaine reinforced the activation of digestive enzymes, ameliorated the intestinal morphology, and enriched the intestinal microbiota of rats with high-salt stressed, which indicates it’s positive effect on intestinal barrier function. Betaine is involved in osmotic regulation of the duodenal epithelium of broiler chicks and has a positive effect on the movement of water across the intestinal epithelial cell in vitro. In addition, betaine supplementation reduced the challenge effect of coccidioides and had a positive effect on the nutrient digestibility and the feed conversion ratio [28, 29]. Dietary betaine can assemble in intestinal tissues and reinforces the structure of intestinal epithelium [30]. However how does betaine improve intestinal function is not clear and needed to explore. It is well-known that tight junction is an essential constituent part of intestinal barrier and has a strongly association with intestinal inflammation. Tight junction proteins Occludin and Claudin-1 are necessary for TJs to maintain intestinal permeability and intestinal barrier function [13]. And the intestinal barrier function is even accommodated in some ways by TJ proteins [31–33]. There are evidences proved that LPS-induced inflammation destroyed the integrity of intestinal epithelial cells and TJs [17]. In the present research, we determined that betaine (0-2 mmol/L) improved the expression of TJ proteins and with the concentration of 2 mmol/L, betaine was able to relive the down- regulation of TJ proteins completely which is induced by LPS. Meanwhile, the results improved that betaine decreased the gene expression of IL-6 which also means betaine was able to withstand inflammatory. TEER has long been considered as a common useful indicator of intestinal epithelial cell permeability [34, 35]. IPEC-J2 cells is a well-accepted intestinal cell line model [36]. The increase of TEER signifies the enhancement of intestinal barrier function and the decrease of permeability. Our results are conformed to it: the TEER of betaine and LPS co-treatment team is recovered almost to the same as ctrl group while LPS decreased the TEER, and betaine alone increased the TEER and enhanced intestinal barrier function. In summary, we can get the information from the results that betaine regulates intestinal barrier function by enhancing the expression of TJ proteins and relieving the LPS-induced destroy of TJs. The results lay a solid mechanism foundation for the daily supplement application of betaine.

## Conclusion

In summary, our research expounded the important regulatory role of betaine in intestinal epithelial barrier function. Different concentrations of betaine improved the expression of TJ proteins Occludin and Claudin-1. With the concentration of 2 mmol/L, betaine recovered the LPS-induced inflammation, the down- regulation of TJ proteins and enhanced the intestinal epithelial barrier function. Therefore, betaine, a non-toxic natural compound, is a useful nutrient supplement to regulate intestinal barrier function and prevent intestinal inflammation.

## Methods

### Materials

Betaine and LPS were purchased from Sigma-Aldrich (Saint Louis, USA) and dissolved in PBS to prepare stock solutions with concentrations of 100 mmol/L and 1 mg/mL respectively. Occludin (Catalog number: #91131) and Claudin-1 (Catalog number: #13995) were obtained from Cell Signaling Technology (Shanghai, China.). All the reagents were stored at − 20 °C.

### Cell culture

IPEC-J2 cells were obtained from Animal Nutrition & Human Health Laboratory, Hunan Normal University. IPEC-J2 cells were cultured in DMEM with 10% of FBS and 1% of penicillin–streptomycin (Hyclone, Logan, UT, USA) at 37 °C, 5% of CO_2_. The medium was refreshed every second day.

### Western blot

Western blot (WB) was used to evaluate the expression of TJ proteins in IPEC-J2 cells. The experimental steps are consistent with regular procedure. First, polyacrylamide gel was prepared and then electrophoresis after adding the required samples. Next, the PVDF membrane was covered by 5% milk for an hour after western transfered, and then the membranes were washed with PBS for three times and incubated with primary antibodies overnight. Next day, the PVDF membranes were washed for six times and then incubated with second- antibody for an hour. Development was performed by chemiluminescence equipment after washing it again for three times and the pictures were measured by Image J.

### Morphological observation of cells

IPEC-J2 cells were inoculated 5 × 10^5^ per well in 6-well plate and cultured for 2 days (the culture medium was replaced 24 h after the inoculation), then the cells were treated by ctrl, LPS, LPS + betaine and betaine alone for 24 h. After that, the culture medium was outwelled and the cells were washed with PBS for two times. The pictures were taken under the light contrast microscopy (Leica, DFC450C, Wetzlar, Germany) in the end.

### Immunofluorescence

IPEC-J2 cell monolayers were incubated and cultured in 6-well plates and each plate has a glass slide where the cells attached to. Then the cells were treated with ctrl, LPS, LPS + betaine and betaine for 24 h. Cells were fixed with paraformaldehyde (4%) no less than 20 min after washing twice with PBS (10mins each). Then cells were blocked for 30 min with bovine serum albumin (BSA 1%) after washing it again and then incubated with Claudin-1 overnight. The second day the cells were washed once again and incubated with second- antibody for 2 h. Cells were washed for another 30 min and then covered with the DAPI dye solution for 10 min. The inverted fluorescence microscope was used to take the microscopic images of the cells.

### RT-PCR

Reverse transcriptase-polymerase chain reaction was used to detect the expression of relative mRNA. First cDNA was extracted by conventional methods, and the PCR system was composed by 5.0 μL of Green qPCR Mix, 0.3 μL of Forward primer and Reverse primer (the sequences of primers shown in Table 1), 4.2 μL of double distilled water, and 0.2 μL of cDNA. The final volume was 10 μL. Then the housekeeping gene GAPDH was used as the standard control, and each sample was repeated for three times, then Real Master Mix SYBP ROX (5′ Prime) was used for quantitative real-time PCR.

**Table 1:** Sequences of Primers

Gene	Forward primer (5′–3′)	Reverse primer (5′–3′)
GAPDH	GAAGGTCGGAGTGAACGGAT	CTGGCATTGACTGGGGTCAT
Occludin	TTGCCTGGGACGAGGCTATG	ATCCCTTTGCTGCTCGTGGA
IL-6	TGGATAAGCTGCAGTCACAG	ATTATCCGAATGGCCCTCAG

### TEER

The 24-hole transwell plate (Costar, Coring Inc., NY, USA) was infiltrated with DMEM culture medium for 2 h or overnight before inoculating IPEC-J2 cells (1 × 10^5^ cells per cm^2^). After being attached, cells were differentiated in the culture medium without serum for 7–10 days. When the resistance value reaches the desired value, the cells were treated with ctrl, LPS, LPS + betaine, betaine respectively. The way to measure the cell integrity is TEER, which used an epithelial voltage ohmmeter with chopstick electrodes (Millicell ERS-2, EMD Millipore, Billerica, MA). TEER measurements were operated in gnotobasis. The plate was taken out of the 37 °C cell incubator and put in the operating floor for at least 30 min, the electrodes were disinfected by 70% ethanol and then dried in the air for 30 s. Then the electrodes were douched with culture medium, immersed the electrodes and make sure the shorter electrode is in the insert of the plate and the longer electrode is located between the outer wall and the filter insert. Ensure all the electrode tips are absolutely immersed by solution and the shorter one does not come into contact with the cells which are growing on the insert. And record the resistance value of 12, 24, 36, 48, 72 h.

### Statistical analysis

All the statistical data were analyzed by the software GraphPad Prism version6. Data were expressed as mean ± SD and relative gene expressions were transformed into natural logarithm scale. One-way and two-way ANOVAs were used to compare the differences between different treatments. A *p*-value 0.05 was considered statistically significant.

## Data Availability

The datasets used and/or analysed during the current study are available from the corresponding author on reasonable request.

## Abbreviations

IPEC-J2: Porcine intestinal epithelial cells
LPS: Lipopolysaccharide
TEER: Transepithelial electrical resistance
TJs: Tight junctions
